# Electrospinning of Grooved Polystyrene Fibers: Effect of Solvent Systems

**DOI:** 10.1186/s11671-015-0949-5

**Published:** 2015-05-27

**Authors:** Wanjun Liu, Chen Huang, Xiangyu Jin

**Affiliations:** Engineering Research Center of Technical Textiles, Ministry of Education, College of Textiles, Donghua University, No. 2999 North Renmin Road, Songjiang, Shanghai, 201620 China

**Keywords:** Electrospinning, Grooved nanofibers, Polystyrene, Solvent systems

## Abstract

Secondary surface texture is of great significance to morphological variety and further expands the application areas of electrospun nanofibers. This paper presents the possibility of directly electrospinning grooved polystyrene (PS) fibers using both single and binary solvent systems. Solvents were classified as low boiling point solvent (LBPS): dichloromethane (DCM), acetone (ACE), and tetrahydrofuran (THF); high boiling point solvent (HBPS): *N*,*N*-dimethylformamide (DMF) and cyclohexanone (CYCo); and non-solvent (NS): 1-butanol (BuOH). By the systematic selection and combination of these solvents at given parameters, we found that single solvent systems produced non-grooved fibers. LBPS/DMF solvent systems resulted in fibers with different grooved textures, while LBPS/CYCo led to fibers with double grooved texture. Grooved fibers can also be fabricated from LBPS/LBPS, NS/LBPS, and NS/HBPS systems under specific conditions. The results indicated that the difference of evaporation rate (DER) between the two solvents played a key role in the formation of grooved texture. The formation of this unique texture should be attributed to three separate mechanisms, namely void-based elongation, wrinkle-based elongation, and collapsed jet-based elongation. Our findings can serve as guidelines for the preparation of ultrafine fibers with grooved secondary texture.

**Electronic supplementary material:**

The online version of this article (doi:10.1186/s11671-015-0949-5) contains supplementary material, which is available to authorized users.

## Background

Electrospinning is a versatile nanofiber production technique using electrical force to stretch a charged polymer solution jet coupled with solvent evaporation and subsequent harvesting of solidified or semi-solidified nanofibers [1, 2]. Electrospun nanofibers are gaining increasing attention due to their distinct properties such as high specific surface area, ease of functionality, variety of morphology and structure, and high porosity and interconnected pores of their assembled nonwovens, which allow them to be investigated and applied to various areas, such as tissue engineering [3, 4], sensors [5], filtration [6–8], and self-cleaning surfaces [9, 10].

Proof has demonstrated that the properties and behavior of nanofibers could be greatly enhanced or altered when their secondary morphologies and structures can be precisely regulated. Lin et al. [11] fabricated self-crimping bicomponent nanofibers using side-by-side electrospinning from polyacrylonitrile and elastomeric polyurethane, which could be used as chemical sensors and nanotweezers. In addition, Lin et al. [12] found that the specific surface of polystyrene (PS) fibers electrospun from 5 wt.% PS solution (tetrahydrofuran (THF)/*N*,*N*-dimethylformamide (DMF), 1:3) was as high as 54.92 m^2^ g^−1^ because of the porous structure, while the counterpart from 30 wt.% PS/THF was only 0.98 m^2^ g^−1^. It has been demonstrated [13–15] that the performance was dramatically improved when porous fibers were employed as absorption materials. Jiang et al. [10] reported that lotus-leaf-like superhydrophobic surfaces with water contact angles (CA) larger than 150° were fabricated using a porous microsphere/nanofiber composite film. Moreover, Ding et al. [5] indicated that porous fibers can also perform well as ultrasensitive sensors.

Most electrospun fibers are cylindrical ones with smooth surface. Although there are some reports on different secondary morphologies, such as self-crimping [11], core-shell [16–18], hollow [19], and porous [13–15, 10, 5, 20], all the textures lack good alignment. Aligned surface texture has been proved to be of great significance to the morphological variety and further expands the application areas of electrospun nanofibers. Xue et al. [21] reported superhydrophobic electrospun POSS-PMMA fibers with highly ordered surface structure using the THF/DMF solvent system, which showed a water contact angle as high as 165° with a sliding angle as low as 6°. Our previous work [22] fabricated cellulose acetate butyrate using the acetone(ACE)/DMF solvent system, which demonstrated that grooved fibers can serve as cues for cell adhesion and proliferation. We [23, 24] also investigated the fabrication of grooved PS fibers using the ACE/DMF and THF/DMF solvent systems.

To the best of our knowledge, few studies have systematically investigated the effect of solvent systems on the formation of grooved texture. Here we selected PS as the model because it can be dissolved by many solvents [25–27]. PS solutions were prepared using both single solvents including dichloromethane (DCM), ACE, THF, DMF, cyclohexanone (CYCo), and 1-butanol (BuOH) and binary solvent systems. The main objectives of this study were to investigate the feasibility of fabricating grooved fibers from various solvent systems, to identify the key factors to form grooved fibers, and to figure out the formation mechanism of grooved texture. The results can provide guidelines for the preparation of ultrafine fibers with grooved secondary texture.

## Methods

### Chemicals and Materials

PS (Mw = 350,000 g/mol) was purchased from Sigma-Aldrich, Inc. DCM, ACE, THF, DMF, CYCo, and BuOH were purchased from Shanghai Chemical Reagents Co., Ltd., China. All materials were used without further purification.

### Electrospinning

PS solution was placed into a syringe with an internal diameter of 0.7 mm, which was mounted on a syringe pump (single-syringe infusion pump KDS 100, KD Scientific Inc., Holliston, USA). A high-voltage supplier (high-voltage direct-current power supply, DW-P503-2ACDE, Tianjin Dongwen Co., Ltd., Tianjin, China) was connected to the syringe needle. In this study, the solvent ratio was the volume ratio, and the solution concentration was weight/volume (g/ml). Electrospinning parameters were selected based on our previous study [24]. Specifically, relative humidity (RH), temperature, collecting distance, feeding rate, and applied voltage were kept at 60 %, 22 °C, 15 cm, 1.5 ml/h, and 12 kV, respectively.

PS solutions with different concentrations were prepared using both single and binary solvent systems. The single solvent systems were DCM, ACE, THF, DMF, CYCo, and BuOH, but ACE and BuOH cannot dissolve PS. The binary solvent systems were DCM/ACE, DCM/THF, ACE/THF, DCM/DMF, ACE/DMF, THF/DMF, DCM/CYCo, ACE/CYCo, THF/CYCo, DMF/CYCo, BuOH/DCM, BuOH/ACE, BuOH/THF, BuOH/DMF, and BuOH/CYCo. All the binary solvent systems were tried using different solvent ratios (3:1, 2:1, 1:1, 1:2, 1:3). However, we failed to prepare fully dissolved 15 % PS solutions when using DCM/ACE (1:3), BuOH/DCM (3:1, 2:1), BuOH/ACE (3:1, 2:1, 1:1, 1:2, 1:3), BuOH/THF (3:1, 2:1), BuOH/DMF (3:1, 2:1), and BuOH/CYCo (3:1, 2:1) solvent systems.

### Characterization

The surface morphology and cross section of the as-spun PS nanofibers were observed by scanning electron microscopy (SEM) (TM-3000 and S-4800, Hitachi Ltd., Japan).

## Results and Discussion

In this study, we investigated the effect of solvent systems using both single and binary solvent systems. Solvents involved can be classified as low boiling point solvent (LBPS): DCM, ACE, and THF; high boiling point solvent (HBPS): DMF and CYCo; and non-solvent (NS): BuOH. Their basic properties (i.e., boiling point, vapor pressure, viscosity, electrical conductivity, dielectric constant, and surface tension) are listed in Table 1.

**Table 1:** Typical properties of the solvents used in this work [30]

Solvent	Boiling point (°C)	Vapor pressure (kPa, 20 °C)	Viscosity (mPa s, 20 °C)	Electrical conductivity (S cm^−1^, 25 °C)	Dielectric constant	Surface tension (mN/m)
DCM	39.75	46.5	0.425	4.30 × 10^−11^	9.10	28.12
ACE	56.12	24	0.316	5.8 × 10^−08^	20.70	23.7
THF	66	19.07	0.55	4.5 × 10^−05^	7.58	26.4
BuOH	117.7	0.73	2.95	9.12 × 10^−9^	17.1	24.6
DMF	153.0	0.36	0.802 (25 °C)	6.0 × 10^−8^	36.71	35.2
CYCo	155.65	0.5	2.2	5 × 10^−8^	18.3	34.50

### Single Solvent Systems

From our initial attempts, DCM, ACE, THF, DMF, CYCo, and BuOH were used as single solvent systems to prepare 15 % PS solutions. Generally, DCM, THF, DMF, and CYCo led to clear PS solutions, but BuOH was unable to dissolve PS pellets and we refer to it as NS. While ACE was also unable to dissolve PS pellets, it resulted in the swelling and softening of PS. Further attempt indicated that even 1 % PS pellets could not be fully dissolved by ACE, and here we refer to ACE as a poor solvent and classify it under the LBPS group. Consequently, PS solutions using DCM, THF, DMF, and CYCo as single solvent systems were successfully electrospun. For DCM and THF, it should be noted that the electrospinning process was unstable and often interrupted by needle clogging due to the fast evaporation of the solvent.

**Fig. 1:**
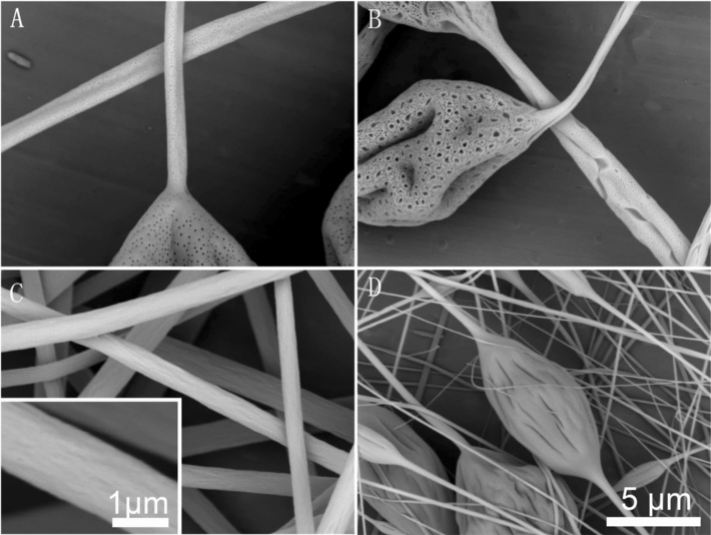
Representative pictures of samples fabricated by electrospinning of 15 % PS solutions from different single solvent systems. **a** DCM, **b** THF, **c** DMF, and **d** CYCo

Figure 1 shows the representative pictures of samples fabricated by electrospinning 15 % PS solutions from different single solvent systems. Only DMF was found to produce beaded free fibers with rough surface, which should be attributed to its higher dielectric constant and electrical conductivity, while other solvents produced beaded fibers with different secondary morphologies. Specifically, DCM led to small surface pores (less than 100 nm) uniformly distributed on the beads and fibers. THF resulted in irregular large pores (about 1 μm in diameter or length) on the beads or elongated large pores on the fibers surrounded by small pores (less than 100 nm). CYCo produced wrinkled beads and smooth fibers. The formation of surface pores from DCM and THF should be ascribed to thermally induced phase separation [24, 28]. The rough surface and wrinkled beads that resulted from DMF and CYCo should be attributed to buckling instability [29] and elongation by electrical force [24].

We also tried to increase the concentration of the PS/CYCo solution. However, even 30 % was still not high enough to produce beaded free fibers (Additional file 1: Figure S1). Further increasing the concentration resulted in a too viscous solution to stir. It should be mentioned that the PS/CYCo solution was much more viscous than the other single solvent systems. The resultant beaded fibers should be attributed to the higher viscosity and surface tension as well as lower electrical conductivity and dielectric constant of CYCo [25]. In addition, the freshly collected fibers were “wet,” and such a phenomenon was not observed from the PS/DMF solution, indicating that the evaporation rate of CYCo is much slower in spite of the similar boiling point and vapor pressure of DMF and CYCo.

### Binary Solvent Systems

Binary solvent systems investigated here can be generally classified as LBPS/LBPS, LBPS/HBPS, HBPS/HBPS, NS/LBPS, and NS/HBPS, which are all the possible combinations of LBPS, HBPS, and NS.

Based on the evolution trend of resultant fibers, the following results are presented as LBPS/LBPS, LBPS/DMF, (LBPS and HBPS)/CYCo, and BuOH/(LBPS and HBPS). In binary solvent systems, solvent ratio is a key factor to the formation of grooved texture. Herein, the solvent ratios investigated were 3:1, 2:1, 1:1, 1:2, and 1:3. Table 2 summarizes the performance of all the binary solvent systems employed in this study. Overall, needle clogging was a common problem in LBPS/LBPS solvent systems, while LBPS/HBPS, LBPS/HBPS, and NS/HBPS performed well without needle clogging except for ACE/CYCo (3:1, 2:1). Electrospinnability was poor for NS/LBPS solvent systems except for BuOH/THF (1:1, 1:2, 1:3).

**Table 2:** Electrospinnability of solutions using different binary solvent systems (\, −, +, ++, +++, and ++++ represent no solution prepared, unelectrospinnable, severe needle clogging, medium needle clogging, occasional needle clogging, and no needle clogging, respectively)

Solvent systems	3:1	2:1	1:1	1:2	1:3
DCM/ACE	++	++	++	++	\
DCM/THF	++	++	++	++	++
ACE/THF	++	++	++	++	++
DCM/DMF	++++	++++	++++	++++	++++
ACE/DMF	++++	++++	++++	++++	++++
THF/DMF	++++	++++	++++	++++	++++
DCM/CYCo	+++	+++	+++	+++	+++
ACE/CYCo	-	++	++++	++++	++++
THF/CYCo	+++	+++	++++	++++	++++
DMF/CYCo	++++	++++	++++	++++	++++
BuOH/DCM	\	\	+	+	+
BuOH/ACE	\	\	\	\	\
BuOH/THF	\	\	++++	++++	+++
BuOH/DMF	\	\	++++	++++	++++
BuOH/CYCo	\	\	++++	++++	++++

#### LBPS/LBPS

In this case, DCM/ACE, DCM/THF, and ACE/THF were employed as binary solvent systems. 15 % PS solutions were successfully prepared using these solvent systems with different ratios except that PS cannot be fully dissolved in DCM/ACE (1:3). We further tried to prepare 10 % PS solution (DCM/ACE, 1:3), which resulted in laminar solution. As ACE is a poor solvent to PS, the solubility of DCM, ACE, and THF to PS could be judged in the following order: THF > DCM > ACE.

**Fig. 2:**
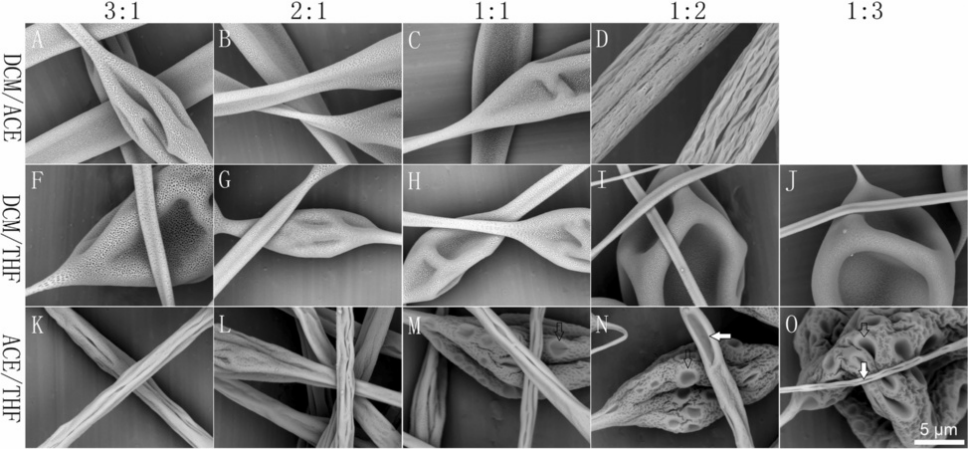
Representative pictures of samples fabricated by electrospinning of 15 % PS solutions from LBPS/LBPS solvent systems with different solvent ratios (3:1, 2:1, 1:1, 1:2, 1:3). **a**–**d** DCM/ACE, **f–j** DCM/THF, and **k–o** ACE/THF

As illustrated in Fig. 2, grooved fibers were obtained using the DCM/ACE and ACE/THF binary solvent systems, both of which contained the poor solvent ACE, while DCM/THF produced only porous beaded fibers. The grooved texture was also influenced by solvent ratio. DCM/ACE allowed for the fabrication of grooved fibers when the ratio was only 1:2, while ACE/THF can produce grooved fibers from all the solvent ratios investigated.

#### LBPS/DMF

**Fig. 3:**
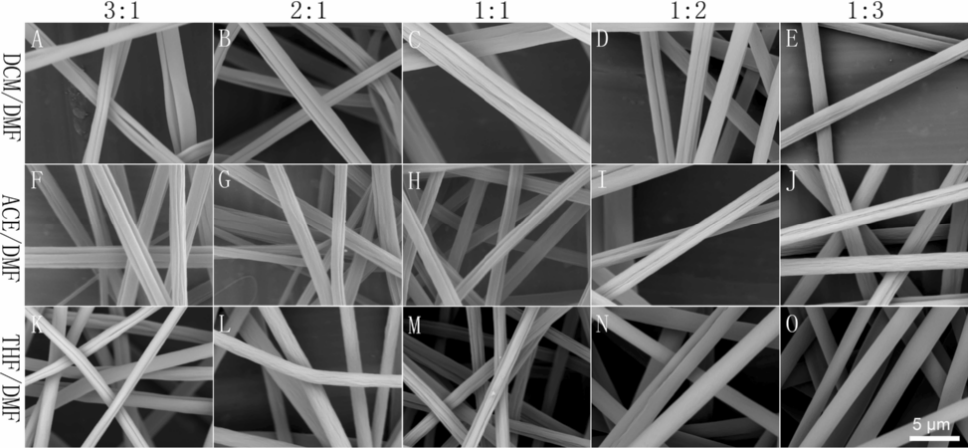
Representative pictures of samples fabricated by electrospinning of 15 % PS solutions from LBPS/DMF solvent systems with different solvent ratios. **a–e** DCM/DMF, **f–j** ACE/DMF, and **k–o** THF/DMF

In this case, DCM/DMF, ACE/DMF, and THF/DMF were employed as binary solvent systems. Here grooved texture was significantly apparent, as shown in Fig. 3. DCM/DMF tended to produce multi-grooved fibers when the ratio was equal to or higher than 1:1, while single grooved texture was more frequently found when the ratio was less than 1:1. ACE/DMF was similar to DCM/DMF. It is noticeable that THF/DMF resulted in single grooved fibers when the solvent ratio was 3:1, fibers with many small grooves distributed along the axis of fibers were obtained when the ratio was 2:1, and a solvent ratio of 1:1 led to typical multi-grooved texture, while a solvent ratio less than 1:1 tended to form smooth fibers.

#### (LBPS and HBPS)/CYCo

**Fig. 4:**
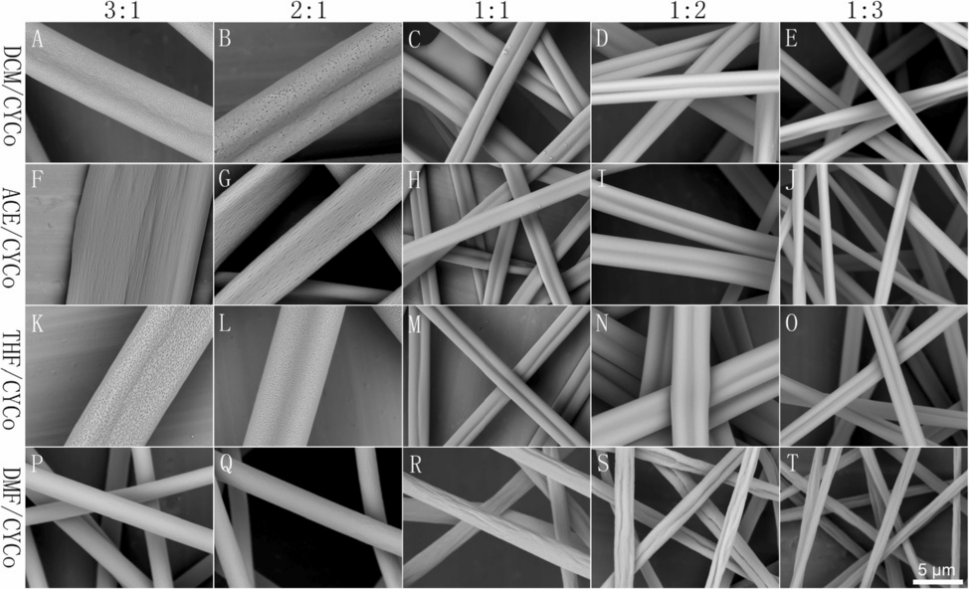
Representative pictures of samples fabricated by electrospinning of 25 % PS solutions from (LBPS and HBPS)/CYCo solvent systems with different solvent ratios (3:1, 2:1, 1:1, 1:2, 1:3). **a–e** DCM/CYCo, **f–j** ACE/ CYCo, **k–o** THF/ CYCo, and **p–t** DMF/CYCo

In this case, DCM/CYCo, ACE/CYCo, THF/CYCo, and DMF/CYCo were employed as binary solvent systems. As shown in Additional file 1: Figure S2, 15 and 20 % PS solutions (solvent ratio, 1:1) resulted in beaded fibers except for ACE/CYCo. When the concentration increased to 25 and 30 %, beaded free fibers were generated, so 25 % was selected due to its lower viscosity. As illustrated in Fig. 4, CYCo led to distinctly different morphologies of PS fibers. Overall, LBPS/CYCo contributed to double grooved fibers, and a solvent ratio higher than 1:1 resulted in double grooved fibers with porous surface because of thermally induced phase separation. It should be noted that the PS solution from ACE/CYCo with a solvent ratio of 3:1 was unable to be electrospun. A solvent ratio equal to or less than 1:1 produced double grooved fibers with smooth surface. In contrast, DMF/CYCo (HBPS/CYCo) formed cylindrical fibers, and a solvent ratio higher than 1:1 resulted in smooth fibers, while that equal to or less than 1:1 led to grooved fibers.

#### NS/(LBPS and HBPS)

**Fig. 5:**
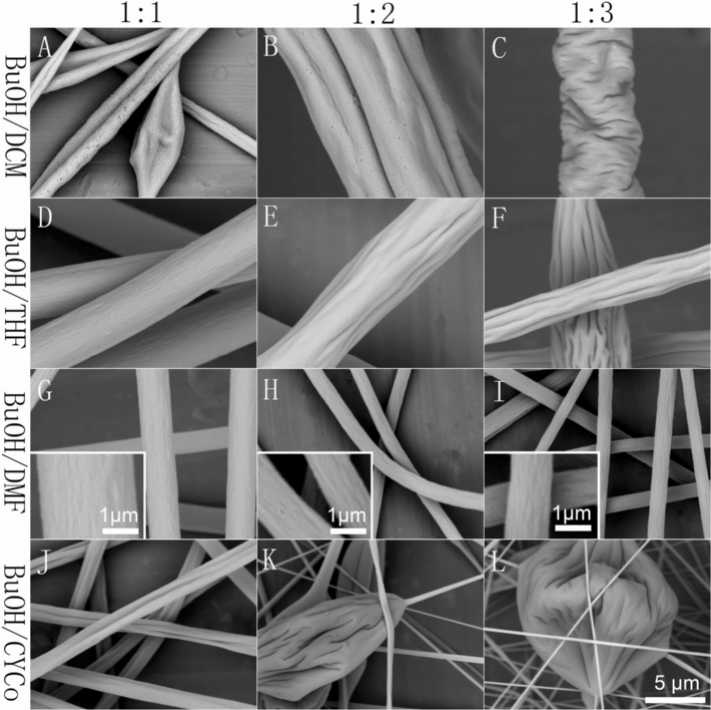
Representative pictures of samples fabricated by electrospinning of PS solutions from BuOH/(LBPS and HBPS) solvent systems with different solvent ratios (1:1, 1:2, 1:3). **a** 10 %, BuOH/DCM. **b**, **c** 15 %, BuOH/DCM. **d–f** 15 %, BuOH/THF. **g–i** 15 %, BuOH/DMF. **j–l** 15 %, BuOH/CYCo

To prepare PS-NS/(LBPS or HBPS) solutions, we selected BuOH because it is miscible to other solvents. Here BuOH/DCM, BuOH/ACE, BuOH/THF, BuOH/DMF, and BuOH/CYCo were employed as binary solvent systems. Similar to the previous procedure, we also prepared 15 % PS solutions with different solvent ratios. PS cannot be dissolved in BuOH/ACE at any ratio as PS is non-soluble to both solvents. For other systems, a solvent ratio higher than 1:1 cannot form a clear solution except for BuOH/DCM (1:1), but a lower concentration solution (10 % PS, BuOH/DCM, 1:1) was successfully prepared and electrospun (Fig. 5a), which resulted in double grooved fibers with surface pores.

More interestingly, NS/(LBPS and HBPS) systems also produced different types of grooved texture (Fig. 5). For BuOH/DCM, the electrospinning process was highly unstable and often interrupted by severe needle clogging. However, the 1:1 solvent ratio produced double grooved fibers with surface pores and beads, while 1:2 resulted in grooved fibers and 1:3 led to a wrinkled surface. In this case, the grooved and wrinkled morphologies are both caused by buckling instability under different driving forces. In other words, grooved texture will be formed when the driving force is sufficient, and a lower driving force resulted in wrinkled texture. BuOH/THF also produced grooved fibers. The 1:1 solvent ratio resulted in fibers with small grooves on the surface, and a lower solvent ratio led to more distinct grooved texture. BuOH/DMF contributed to fibers with rough surface. BuOH/CYCo produced double grooved fibers when the ratio is 1:1, whereas a lower ratio resulted in beaded fibers.

### Further Discussion on the Formation Mechanism of Grooved Texture

Table 3 summarizes the difference of boiling point (DBP) of binary solvent systems and morphologies of fibers electrospun from these systems. Among the solvent systems employed, single solvent systems produced non-grooved fibers, while binary solvent systems tended to produce fibers with well-aligned grooved texture. Specifically, LBPS/DMF systems produced fibers with multi-grooved texture, while LBPS/CYCo systems led to fibers with double grooved texture. In particular, grooved fibers can also be generated from LBPS/LBPS and NS/(LBPS and HBPS) under specific conditions.

**Table 3:** DBP of binary solvent systems and morphologies of fibers electrospun from these systems (\ and − represent no solution prepared and unspinnable, respectively)

Solvent systems	DBP (°C)	3:1	2:1	1:1	1:2	1:3
DCM/ACE	16.37	Pores + beads	Pores + beads	Pores + beads	Grooves	\
DCM/THF	26.25	Pores + beads	Pores + beads	Pores + beads	Pores + beads	Pores + beads
ACE/THF	9.88	Grooves	Grooves + beads	Grooves + beads	Grooves + beads	Grooves + beads
DCM/DMF	113.25	Grooves	Grooves	Grooves	Single groove	Single groove
ACE/DMF	96.88	Grooves	Grooves	Grooves	Single groove	Single groove
THF/DMF	87	Single groove	Grooves	Grooves	Smooth	Smooth
DCM/CYCo	115.9	Double grooves	Double grooves	Double grooves	Double grooves	Double grooves
ACE/CYCo	99.53	-	Double grooves	Double grooves	Double grooves	Double grooves
THF/CYCo	89.65	Double grooves	Double grooves	Double grooves	Double grooves	Double grooves
DMF/CYCo	2.65	Smooth fibers	Smooth fibers	Grooves	Grooves	Grooves
BuOH/DCM	77.95	\	\	Double grooves	Grooves	Wrinkles
BuOH/ACE	61.58	\	\	\	\	\
BuOH/THF	51.7	\	\	Grooves	Grooves	Grooves
BuOH/DMF	35.3	\	\	Rough fibers	Rough fibers	Rough fibers
BuOH/CYCo	37.95	\	\	Double grooves	Smooth fibers + beads	Smooth fibers + beads

**Fig. 6:**
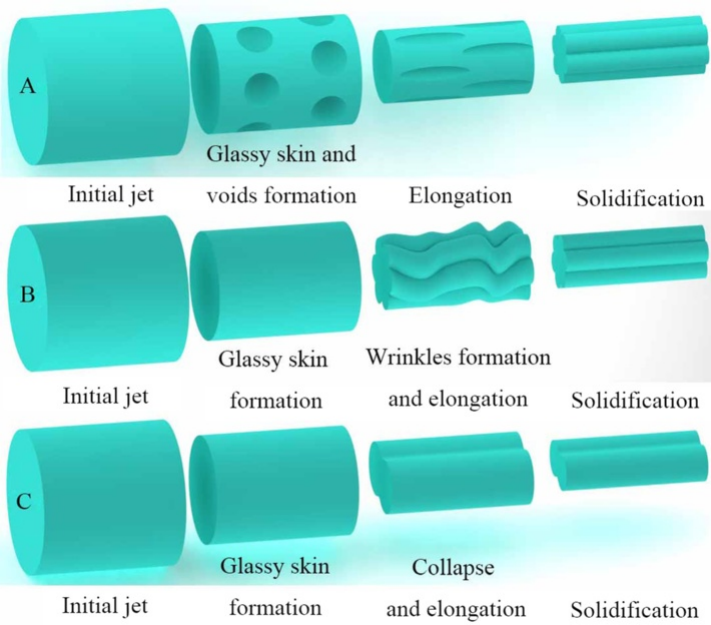
Illustration of grooved texture formation mechanism. **a** Mechanism I, **b** mechanism II, and **c** mechanism III

Our previous research indicated that void-based elongation (Fig. 6a, mechanism I) and wrinkle-based elongation (Fig. 6b, mechanism II) were the two main formation mechanisms of grooved texture [22, 24]. Here we point out that enough difference of evaporation rate (DER) between the two solvents in the binary solvent system is essential to the formation of grooved texture. A solvent with a high boiling point is less volatile and usually has lower evaporation rate than a solvent with a low boiling point, so DER can be generally indicated by DBP. For mechanism I, a highly volatile solvent can facilitate the formation of glassy skin and voids due to its fast evaporation and phase separation, while a low volatile solvent keeps the jet “wet” and stretchable, which allows the subsequent elongation of glassy skin and voids into grooved texture. For mechanism II, glassy skin is formed similar to mechanism I; while the difference is the formation of wrinkles and following elongation into grooved texture, the formation of interior pores contributes to shrinkage of the polymer jet, resulting in the wrinkled surface.

For LBPS/LBPS systems, as we can find obvious large pores with a diameter of a few micrometers on the surface of beads (empty arrows in Fig. 2 m–o), we can also find defects of grooved texture which should be ascribed to the insufficient elongation (white arrows in Fig. 2n, o). So the grooved fibers formed from ACE/THF system should be attributed to mechanism I (Fig. 2 k–o). For DCM/ACE and ACE/THF solvent systems, even though the boiling point and vapor pressure between ACE and THF, DCM are similar, ACE should be the most volatile solvent among ACE, THF, and DCE due to its poor solubility to PS, which enables enough DER to form grooved fibers. In addition, the higher boiling point of DCM results in smaller DER between DCM and CE, which should be why non-grooved fibers were electrospun from a ratio higher than 1:2. For the DCM/THF system, the boiling points of DCM and THF are 39.75 and 66 °C, respectively, so the insufficient DER (DBP, 26.25 °C) should be the reason that non-grooved fibers formed.

**Fig. 7:**
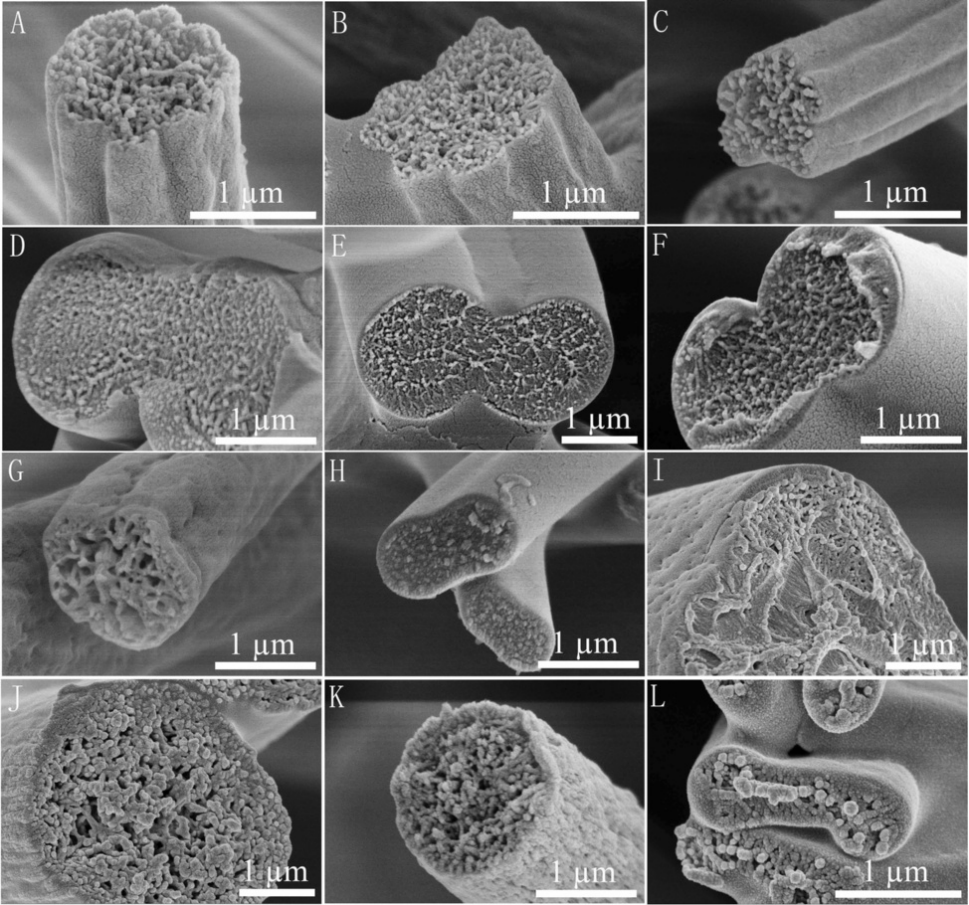
Cross sections of representative PS fibers. 15 % PS solution (solvent ratio 1:1) **a** DCM/DMF, **b** ACE/DMF, and **c** THF/DMF. 25 % PS solution (solvent ratio 1:1) **d** DCM/CYCo, **e** ACE/CYCo, **f** THF/CYCo, **g** DMF/CYCo, and **h** CYCo. 10 % PS solution (solvent ratio 1:1) **i** BuOH/DCM. 15 % PS solution (solvent ratio 1:1) **j** BuOH/THF, **k** BuOH/DMF, and **l** BuOH/CYCo

For LBPS/DMF systems, as the DER was high enough (DBP >87 °C), grooved fibers were routinely electrospun. When the solvent ratio is 1:1, we can confirm the grooved texture by the distinct sawtooth cross section (Fig. 7a–c), and mechanism II should be the predominant formation rule as we can find obvious large pores in the interior structure and a clear thin surface. While for the THF/DMF system, the single grooved texture should be ascribed to mechanism I, which was discussed in our previous paper [24].

For LBPS/CYCo systems, double grooved fibers can be validated by the cross section (Fig. 7d–f), and the interior structure tended to be similar to single CYCo systems (Fig. 7 h). The double grooves produced in the presence of CYCo should be attributed to the formation of glassy skin, subsequent collapse of the jet, and then the elongation into double grooved fibers (Fig. 6 c, mechanism III). In these cases, LBPS played an important role to form a glassy skin due to their fast evaporation, while CYCo was another key factor due to its rather slow evaporation rate and high viscosity, which caused the collapse of the polymer jet. For the DMF/CYCo system, even though grooved fibers were obtained when the solvent ratio was equal to or less than 1:1, the grooved texture was not well organized, which can be confirmed by the cross section (Fig. 7 g). Obviously, the DER between DMF and CYCo is quite small (DBP, 2.65 °C), so this kind of glassy skin was formed later, leaving insufficient time to be elongated into well-aligned grooved texture before reaching the collector.

For BuOH/(LBPS and HBPS) systems, large pores can be found in the interior structure (Fig. 7i–l). The higher DER of BuOH/DCM (DBP, 77.95 °C) and BuOH/THF (DBP, 51.7 °C) and the formation of interior pores contributed to the formation of grooved texture (Fig. 5a, b, e, f), which should be ascribed to mechanism II, while BuOH/DMF led to fibers with rough surface because of the insufficient DER (DBP, 35.5 °C). However, BuOH/CYCo (solvent ratio, 1:1) produced double grooved fibers, which should be attributed to mechanism III. Although the theoretical DBP is only 37.95 °C, the rather slow evaporation rate of CYCo made the actual DER much larger.

## Conclusions

We have demonstrated the feasibility to directly electrospin grooved PS fibers by systematically investigating both single and binary solvent systems at given parameters. We found that single solvent systems produced non-grooved fibers. LBPS/DMF solvent systems generated fibers with different grooved textures, while LBPS/CYCo led to fibers with double grooved texture. Grooved fibers can also be fabricated from LBPS/LBPS, NS/LBPS, and NS/HBPS systems under specific conditions. The results indicated that grooved fibers can be fabricated from binary solvent systems as long as the DER is high enough. The formation of grooved texture should be attributed to three mechanisms, namely void-based elongation, wrinkle-based elongation, and collapsed jet-based elongation.

## Additional file

Additional file 1:
**Supplementary information. A file showing two supplementary figures.**

## Abbreviations

ACE: acetone
BuOH: 1-butanol
CA: water contact angles
CYCo: cyclohexanone
DBP: difference of boiling point
DCM: dichloromethane
DER: difference of evaporation rate
DMF: *N*,*N*-dimethylformamide
HBPS: high boiling point solvent
LBPS: low boiling point solvent
NS: non-solvent
PS: polystyrene
SEM: scanning electron microscopy
THF: tetrahydrofuran
