# Supplementary material for: Electrospinning of Grooved Polystyrene Fibers: Effect of Solvent Systems
Source: Nanoscale Res Lett. 2015 May 27;10:237. doi: 10.1186/s11671-015-0949-5 (PMC4456588; doi:10.1186/s11671-015-0949-5)
Supplement: Additional file 1: — Supplementary information. A file showing two supplementary figures. [file 11671_2015_949_MOESM1_ESM.docx]

**Electrospinning of grooved** **polystyrene fibres: Effect of solvent systems**

Wanjun Liu, Chen Huang, Xiangyu Jin*

Electronic Supplementary Information (ESI) available:


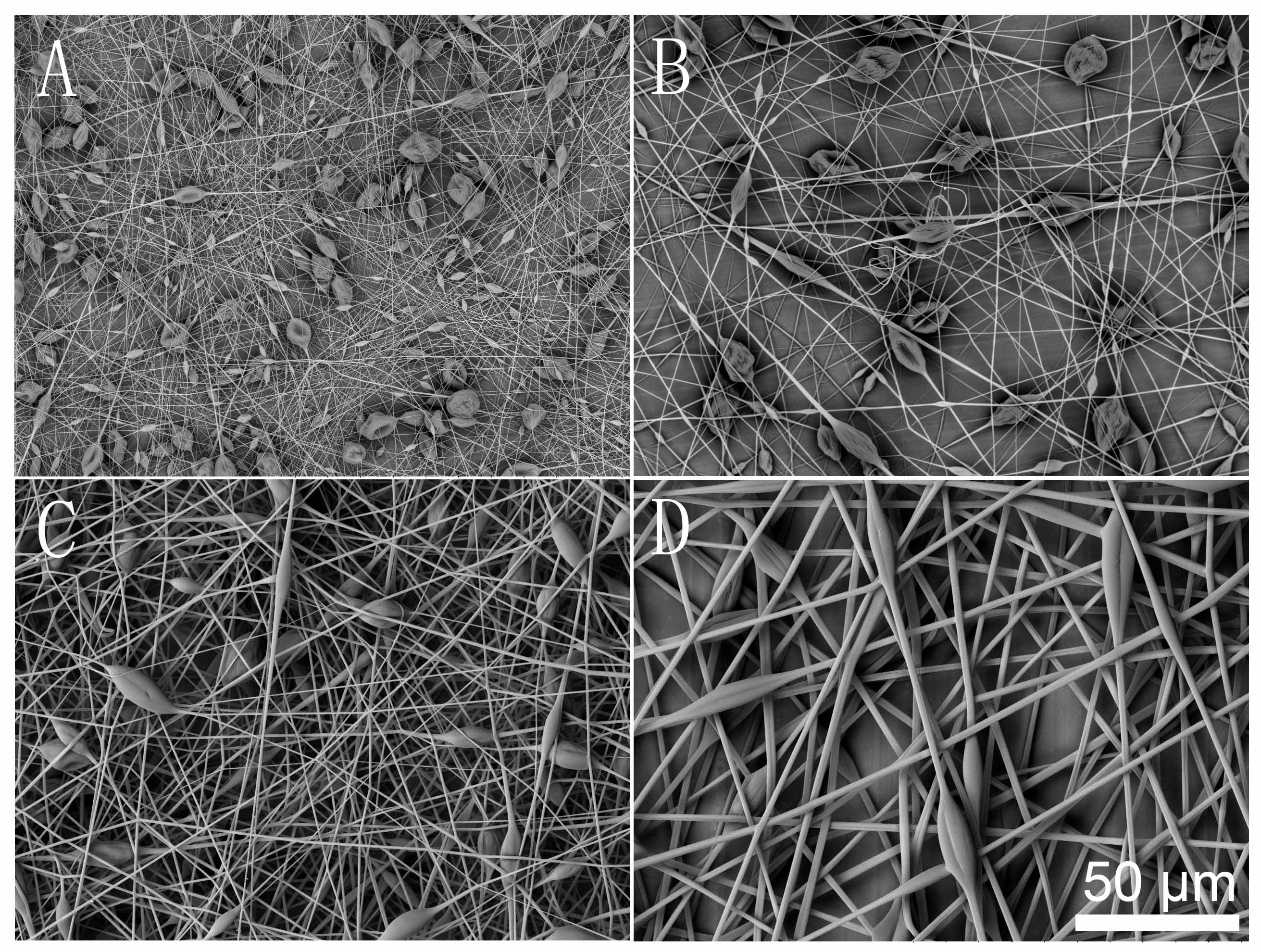


Figure S1 Representative pictures of samples fabricated by electrospinning of PS/CYCo solutions with different concentrations. (A) 15%, (B) 20%, (C) 25%, and (D) 30%


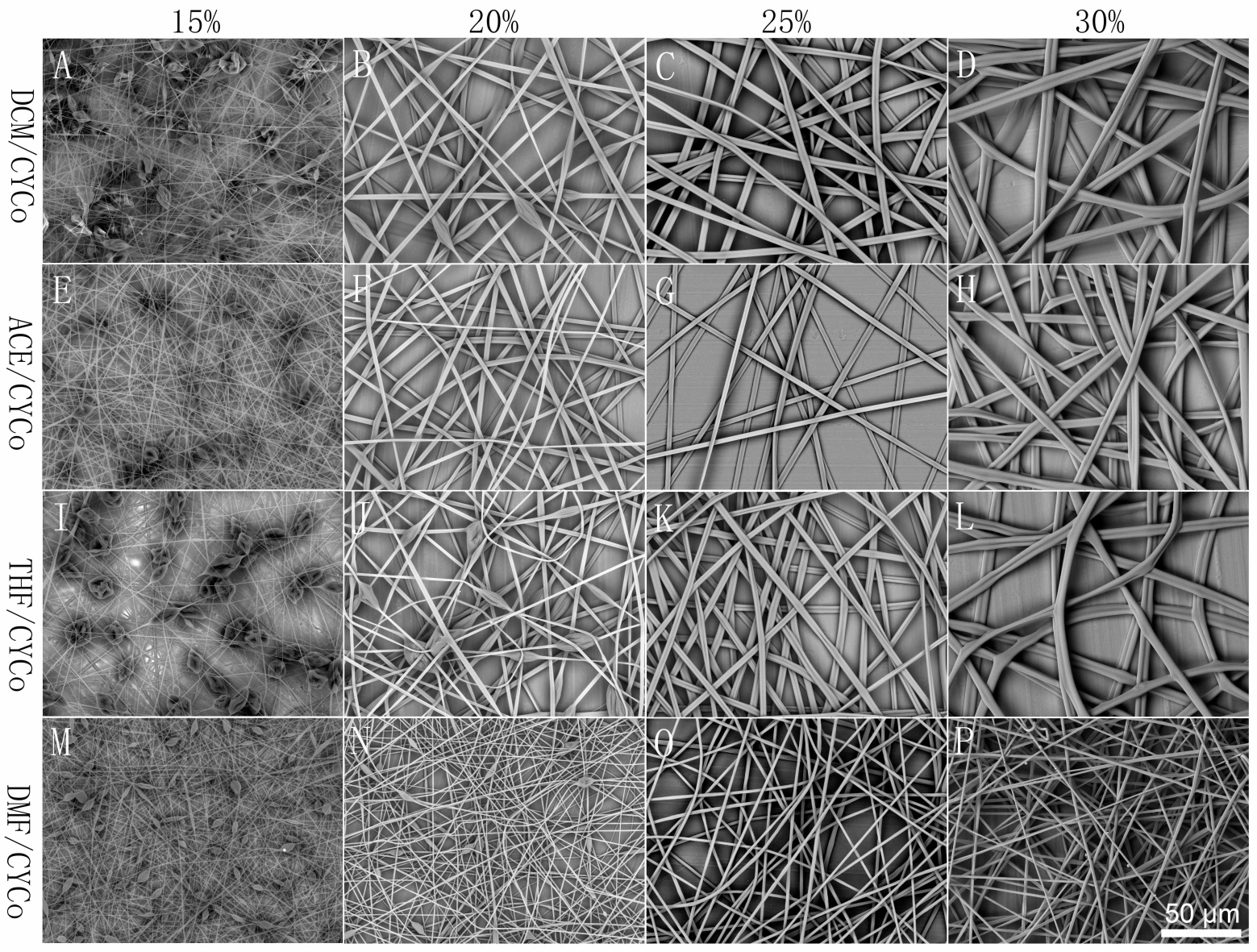


Figure S2 Representative pictures of samples fabricated by electrospinning of PS solutions from (LBPS and HBPS)/CYCo solvent systems with different concentrations (solvent ratio, 1:1). DCM/CYCo (A, B, C, D), ACE/CYCo (E, F, G, H), THF/CYCo (I, J, K, L), and DMF/CYCo (M, N, O, P)
